# Exploring Candidate Genes for Pericarp Russet Pigmentation of Sand Pear (*Pyrus pyrifolia*) via RNA-Seq Data in Two Genotypes Contrasting for Pericarp Color

**DOI:** 10.1371/journal.pone.0083675

**Published:** 2014-01-06

**Authors:** Yue-zhi Wang, Mei-song Dai, Shu-jun Zhang, Ze-bin Shi

**Affiliations:** Institute of Horticulture, Zhejiang Academy of Agricultural Sciences, Hangzhou, Zhejiang Province, China; CNR, Italy

## Abstract

Sand pear (*Pyrus pyrifolia*) russet pericarp is an important trait affecting both the quality and stress tolerance of fruits. This trait is controlled by a relative complex genetic process, with some fundamental biological questions such as how many and which genes are involved in the process remaining elusive. In this study, we explored differentially expressed genes between the russet- and green-pericarp offspring from the sand pear (*Pyrus pyrifolia*) cv. ‘Qingxiang’ × ‘Cuiguan’ F1 group by RNA-seq-based bulked segregant analysis (BSA). A total of 29,100 unigenes were identified and 206 of which showed significant differences in expression level (log_2_fold values>1) between the two types of pericarp pools. Gene Ontology (GO) analyses detected 123 unigenes in GO terms related to ‘cellular_component’ and ‘biological_process’, suggesting developmental and growth differentiations between the two types. GO categories associated with various aspects of ‘lipid metabolic processes’, ‘transport’, ‘response to stress’, ‘oxidation-reduction process’ and more were enriched with genes with divergent expressions between the two libraries. Detailed examination of a selected set of these categories revealed repressed expressions of candidate genes for suberin, cutin and wax biosynthesis in the russet pericarps.Genes encoding putative cinnamoyl-CoA reductase (CCR), cinnamyl alcohol dehydrogenase (CAD) and peroxidase (POD) that are involved in the lignin biosynthesis were suggested to be candidates for pigmentation of sand pear russet pericarps. Nine differentially expressed genes were analyzed for their expressions using qRT-PCR and the results were consistent with those obtained from Illumina RNA-sequencing. This study provides a comprehensive molecular biology insight into the sand pear pericarp pigmentation and appearance quality formation.

## Introduction

Terrestrial plants have evolved special epidermal tissues to adapt to the environmental changes. An extracellular lipid layer termed cuticle covering plant primary organs (such as leaves, primary stems, flowers and fruits) plays a central role in the interaction and adaptation of plants to the terrestrial environment, particularly by imparting resistance to desiccation and providing the first barrier to many pathogens [1]. Cuticle consists of wax crystals with two different types, which are the epicuticular waxes consisting exclusively of very-long-chain aliphatics and the intracuticular waxes containing large quantities of pentacyclic triterpenoids, respctively [2]. The epicuticular waxes cover the cuticle properly, which is cutin embedded with intracuticular waxes. Cuticle also contains suberin that is mostly deposited on the inner face of primary cell walls [3]. Cork layer protects the surfaces of both secondary and mature tissues, including healing tissues. The chemical composition of cork is largely clear despite of some contradicts in reports. The most relevant characteristic of cork in its chemical composition is the presence of suberin as the main cell wall component (33% to 50% reported), and the suberin-associated waxes in the suberin complex of the lamellated secondary wall (approximately 5%) [4]. Unlike cuticular waxes that are chemically distinct from cutin monomers, suberin-associated waxes contain components that are closely related to suberin monomers [5]. The cork layer can protect plant organs against dehydration and pathogens, largely due to the suberin and wax waterproofing properties [1], [2], [6]. Except for suberin and waxes, chemicals including lignin, tannins and other phenolic substances are also structural components of the cork cell wall [7]–[9].

Major components of cork and cuticle are closely related in terms of metabolic pathways. Suberin and cutin are polymers of fatty acid (FA) derivatives linked by ester bonds with glycerol. Suberin comprises an aliphatic and an aromatic domain by high levels of hydroxycinnamic acids (predominantly ferulate), fatty alcohols and saturated aliphatics, with chain lengths >C20, while cutin is mostly composed of C16 and C18 ω-hydroxyacid, polyhydroxy acid, epoxyacid and α, ω-dicarboxylic acid (DCA) [3], [5], [10]. The aromatic domain is a polyphenolic substance mostly composed of hydroxycinnamic acid derivatives and is presumably involved in linking the aliphatic domain to cell wall [11], [12]. Like that of suberin and cutin, the synthesis of waxes begins with the general FA synthesis pathway giving rise to long-chain FAs (LCFAs), that the condensation of very-long-chain FAs (VLCFAs) takes place in the endoplasmic reticulum [5], [13]. Waxes are heterogeneous mixture of VLCFAs and their alkane, aldehyde, alcohol, ketone, and ester derivatives, which typically range from C24–C32 in length [13]. Li *et al*. [14] showed that root waxes components can be produced in the aerial cuticle by ectopic expression of a suberin-associated acyltransferase gene (*GPAT5*), which suggests a direct metabolic link between suberin and some root waxes.

Because of their critical roles to plants, biochemical and molecular genetic researches on the biosynthesis of suberin, cutin and waxes have been carried out extensively in the past years [1]. A few studies on enzymatic activities involved in suberization, most of which dealt with the aspect of aromatic metabolism were summarized in Soler *et al*. [15]. Mutant and gain-of-function approaches revealed three families of the enzymes required for the polymer biogenesis: FA oxidases of the CYP86A sub-family; an acyl-activating enzyme of the LACS family; and acyltransferases of the GPAT family [5]. Genome-wide studies from the suberin-rich phellem of cork tree (*Quercus suber*) and potato (*Solanum tuberosum*) [15], [16] and the identification of genes up-regulated after inducing the Arabidopsis transcription factor WIN1/SHINE1 for cutin and waxes biosynthesis [17] provided valuable additional sources of gene candidates. As the second most abundant plant biopolymer after cellulose, lignin is deposited mostly in the secondary cell walls of vascular plants and is essential for water transport, mechanical support and plant pathogen defense [18]. In previous studies, forward and reverse genetic approaches have revealed a number of genes involved in the regulation of lignin biosynthesis [19]–[23]. Global xylem transcript profiling analysis in Arabidopsis and some tree species uncovered a set of candidates involved in the lignin biosynthesis pathway [24]–[28]. Molecular and genetic studies revealed a group of secondary wall-associated NAC domain transcription factors as major switches regulating a cascade of downstream transcription factors, leading to the activation of the secondary wall biosynthetic program [29]. The molecular genetics of regulatory networks in xylem were also summarized in other reviews [18], [30], [31].

PM-localized ATP-binding cassette (ABC) transporters were identified to be involved in cutin/suberin and lignin transport processes [32]–[39]. Null mutations of a gene encoding a half-transporter ABCG11 caused dwarfism, postgenital organ fusions, altered epidermal cell differentiation, reduced cutin and wax contents [32], [35] and altered suberin composition [33]. Studies using bimolecular fluorescence complementation and protein traffic assays *in vivo* demonstrated that ABCG11 formed homodimers and flexible dimer partnerships with other half-transporters like ABCG12/CER5 [36].

Pears (*Pyrus* spp) are widely cultivated as an important fruit tree in the world. Pear pericarps are made up of cuticle lamellae, epidermal cell layer and cork cambium from outside to inside [40]. The cork cambium forms the thick-walled cell layer, i.e. cork layer, in mature fruit pericarps [41]. Though conservative in structure as mentioned above, variations inpericarp color exist in the sand pear (*Pyrus pyrifolia*) germplasm, that are basically russet, green, and intermediate (russet and green). The russet pericarp of sand pear is suggested be caused by the accumulation of cork layer and is supposed as an important horticultural trait in that it can protect the fruit from external stresses caused by diseases, insects, unfavorable weather and shipping. Inoue *et al.* [42] reported a near 3∶1 segregation of russet and non-russet (including green and intermediate) pericarps in the F1 offspring of a cross between two parents with russet pericarps. A near 1∶1 segregation was observed in a F1 population derived from a russet and intermediate color cross. Nevertheless, Kim *et al.* [43] found a normal distribution of pericarp color in the F1 population of *Pyrus pyrifolia* Nakai‘Niitaka’× *Pyrus ussuriensis* Maxim. Obviously, the inheritance of pericarp color is parental genotype dependent, indicating a complex genetic process in regulating the pear pericarp russet pigmentation.

In the current study, a RNA-seq-Based BSA approach was employed to dissect the genetics of pear russet pericarp and to identify genes related to the cuticle and cork layer formation. Large-scale transcriptome sequencing of the pericarp pools of both russet- and green-pericarp offspring from a segregation population of ‘Qingxiang’ × ‘Cuiguan’ were conducted using the Illumina Sequencing platform. A total of 58, 524, 312 ESTs (101 bp in length each) were obtained, assembled into 29,100 unigenes and annotated extensively. Digital expression analysis of these ESTs identified 206 unigenes that were differentially expressed between the two pools. Analysis of the profiling results allowed for identification of discrete sets of candidate genes involved in various aspects of sand pear pericarp pigmentation, including novel candidates for transcriptional regulation, monomers polymerization, transport and deposition of suberin, cutin, waxes and lignin, and phenylalanine biosynthesis. As far as we know, this is the first report to use a global approach to decipher the molecular biology of fruit cork and suberin.

## Materials and Methods

### Plant material and tissue harvesting

Sand pear pericarp (periderm) was collected from 80-d developing fruits of F1 offspring from the cross of ‘Qingxiang’ × ‘Cuiguan’ growing in Yangdu orchard of Zhejiang Academy of Agricultural Sciences (308290 N, 1208150 E, Haining County, Zhejiang Province, China) in June 2012. Fruits were manually collected to avoid any mechanical damage. The periderm tissues were dissected using sterile scalpels. Same amounts of pericarp tissue from each of 10 russet- and 10 green-pericarp offspring were collected and pooled separately for RNA extraction. Tissue samples were immediately frozen in liquid nitrogen until RNA extraction. The 80-d developing fruits were selected for this study because they were at the beginning of the transition from green to russet pericarps, therefore their phellogen and derivative cells were considered actively engaged in the suberin and cork layer biosynthesis and periderm differentiation processes.

### cDNA preparation and sequencing

Total RNA was extracted from 5 g of each pericarp pool grounded in liquid N_2_ using the Plant RNA Purification Reagent (Invitrogen, Inc.). The cDNA libraries were constructed for each pericarp pool using the TruSeq™ RNA Sample Preparation Kit (Illumina, Inc.) according to the manufacturer's instructions. Briefly, the protocol consists of the following steps: Poly-A containing mRNA was purified from 5 µg total RNA using oligo(dT) magnetic beads and fragmented into 200–500 bp using divalent cations at 94°C for 5 min. The cleaved RNA fragments were reverse-transcribed into first strand cDNA using SuperScript II reverse transcriptase (Life Technologies, Inc.) and random primers. After second strand cDNA synthesis, fragments were end repaired, A-tailed and ligated with indexed adapters. The products were purified and enriched by PCR to create the final cDNA libraries. Target bands were harvested through 2% agarose gel electrophoresis and quantified by TBS380 (Picogreen). The tagged cDNA libraries were pooled in equal ratio and used for 101 bp paired-end sequencing in a single lane of the Illumina HiSeq2000 (Majorbio Bio-pharm Technology Co., Ltd, Shanghai, China) with 51 plus 7 cycles.

### Processing and Mapping of Illumina Reads

The RNA-Seq reads generated by HiSeq2000 were initially processed to remove the adapter and primer sequences using the software SeqPrep (https://github.com/jstjohn/SeqPrep). Sequences less than 20-bp long after removing adapters and primers were discarded from further analyses. Low quality bases at the 3′ end were removed using Condetri (http://code.google.com/p/condetri/downloads/detail?name=condetri_v2.0.pl). The reads were then mapped to the pear genome using a spliced aligner called Tophat with default parameters [44]. Release Pbr_v1.0 downloaded from the database of Pear Genome Project (http://peargenome.njau.edu.cn:8004/) allowed the identification of splicing sites involving novel exons and novel intergenic transcripts. As the pear reference genome sequence was given as scaffolds, gene prediction was performed using the software cufflinks (http://cufflinks.cbcb.umd.edu/) [45] to generate a gtf profile (transcripts.gtf) for gene notation and expression quantification. Gene open reading frames were predicted using the software Trinity (http://trinityrnaseq.sourceforge.net/analysis/extract_proteins_from_trinity_transcripts.html). Based on the alignment of these shorter segments, the genomic regions are assembled and searched for spliced alignments. *Transcript Assembly and differential expression detection*.

The SAS (Sequence Alignment/Scaffold) files generated by Tophat were input to the software Cufflinks [45] to assemble the alignments into transfrags (a gtf file of transcripts). Cufflinks constructs a minimum set of transcripts that best describe the RNA-Seq reads. The unit of measurement used by Cufflinks to estimate transcript abundance is Fragments Per Kilobase of exon per Million fragments mapped (FPKM). Cufflinks assigns reads to the assembled isoforms by probability. The two assembly/tissue types of green- (S1) and russet-pericarp pool (S2) generated by Cufflinks were merged with the reference annotation (the gtf profile derived from Pbr_v1.0) by Cuffmerge [45]. The class codes in the cuffmerge output were used to identify novel isoforms, intergenic transcripts and splice junctions.

The FPKM method was used to correct for biases in total gene exon size and to normalize the total short read sequences obtained from each tissue library. Genes with a false discovery rate (FDR) ≤0.001, and estimated absolute log_2_fold change>1 in sequence counts across libraries were considered significantly differentially expressed. Differentially expressed genes that were co-expressed in the two types of pericarp pools were then subjected to cluster analysis using the R program (a language and environment for statistical computing and graphics). The clusters of differently expressed genes were visualized using cummeRbund (http://compbio.mit.edu/cummeRbund/). KEGG metabolic pathway annotation was performed using Kobas software.

### Quantitative Real Time-PCR (qRT-PCR) Analysis

Isolation of total RNA from the S1 and S2 pericarp pools and synthesis of first-strand cDNA were performed as described above. The transcript levels of nine genes were analyzed by quantitative real time-PCR (qRT-PCR) using the ABI 7500 Real-Time PCR System (Applied Biosystems, USA). Each reaction (final volume 20 µl) contained 10 µl 2 × TransStartTM Top Green qPCR SuperMix (TransGen, Beijing), 0.5 µl Passive Reference DyeII(50×), 0.4 µl of each the forward and reverse primers (10 mM), 2 µl of the cDNA template (corresponding to 50 ng of total RNA), and 7 µl of RNase-free water. The reaction mixtures were heated to 94°C for 30 s, followed by 40 cycles at 94°C for 5 s, 55°C for 15 s, and 72°C for 34 s. A melting curve was generated for each sample at the end of each run to monitor the purity of the amplified products.

All gene-specific primers for qRT-PCR were designed using the Primer 6.0 program (PREMIER Biosoft International, Canada) (Table 1). Each assay using the gene-specific primers amplified a single product of correct size with high PCR efficiency (90–110%) [46]. Elongation factor 1 (EF1-a) gene (GenBank accession number: AY338250) was used as the reference (forward primer: 5′-GGTGTGAAGCAGATGATTTG-3′, reverse primer: 5′-TCACCCTCAAACCCAGATAT-3′) [47], [48]. All qRT-PCR results were normalized using the Ct value corresponding to *EF1-a*. The relative expression levels of target genes were calculated with the 2^−ΔΔCT^ method [49]. Values reported represent the average of three biological replicates.

**Table 1:** Primers of selected genes used for qRT-PCR analysis.

Unigene	Predict coding protein	Primer sequence (5′-3′)	Product length (bp)
GALR01005880	cytochrome P450 86A2	F: CGTTCCACAAGACTCCAA	105
		R: CGACTGATTGCGTAGATTG	
GALR01015948	Mal d 1-like	F: TTGGTGGCATCTCCTGAT	93
		R: CTTAACTTGCTCCTCCTTGA	
GALR01006906	glycosyltransferase	F: ACTGGAGGATATGTAGGTAAC	140
		R: GCTTGGTACTTCGCAATC	
GALR01012212	acyltransferase	F: GAATGCCATCGTTGTAAGC	94
		R: TGAGCGGAAGAGACAGTT	
GALR01009736	SAUR family protein	F: TTGAAGAAGCCGAGTCTG	109
		R: TCGCCACAATCATCCATT	
GALR01010946	fatty acyl-CoA reductase 3 isoform 1	F: ACTGTCCGTTACTTACTGTTC	121
		R: CCACCAGCCTCATCACAA	
GALR01016595	fatty acid desaturase 3	F: CGGCAAGGAATGGAGTTAT	117
		R: TGAGGGAAGAGATGATGGAT	
GALR01020254	patellin-3-like	F: AGACTCTTATGGAACACTGT	116
		R: AGCTCACTGGCATCAATT	
GALR01020707	cinnamoyl-CoA reductase	F: CTGTTGCCACCTTCTCAA	108
		R: GTTGCTTATCTTCTGTTGGT	

### Functional annotation

The transcripts with ORFs and no ORFs were compared to the NT, NR, String and KEGG-Gene databases by BlastP and BlastX, respectively. An E-value of 1.0×10^−5^ was taken as the threshold for significance.

### Gene Ontology Classification

The GO functional annotations for the pear transcripts were performed using the software blast2go (http://www.blast2go.com/b2glaunch/start-blast2go). For functional enrichment analysis, the transcripts were classified into the categories of “cellular component”, “molecular function” or “biological process”. GOseq of the R package [50] was used for this purpose and a list of GO-Terms and their corresponding Z-scores were included in the result file. The Z-score for each term was given using the following formula: Z  =  (Sm − μ)*m1/2/δ; where Sm is the mean T-statistic value of a gene annotated with a GO-term, μ is the mean T-statistic value of the entire gene list and δ is the standard deviation of the T-statistic value in the entire gene list. P-values were calculated for each GO-Term from their Z-scores and were corrected for multiple test. A q-value cutoff of 0.05 was used to determine enriched GO-terms.

## Results

### Library Construction and Sequencing

**Figure 1:**
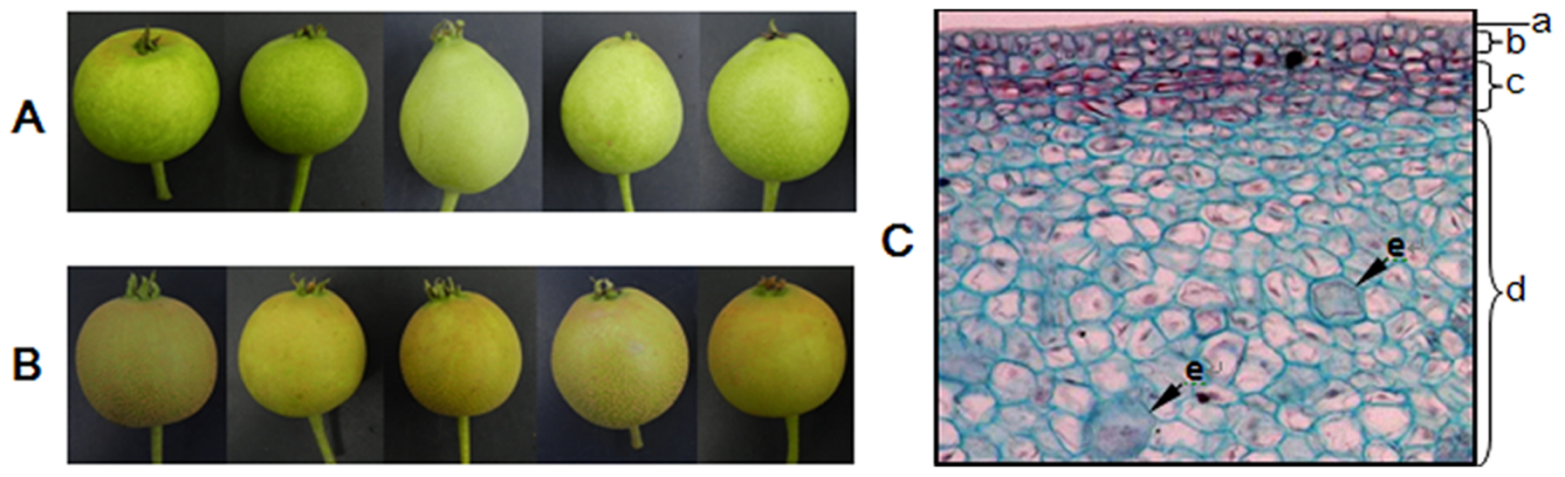
Pericarp pigmentation of sand pear cv. ‘Cuiguan × Qingxiang’ offspring, and microscopic structure of ‘Cuiguan’ young fruit pericarp (green) under 200× magnification. A, green-pericarp fruits at 80 day after flowering (DAP); B, russet-pericarp fruits at 80 DAP; C, a∼e, cuticle layer (a),Epidermal cell layer (b),Cork cambium (c),Parenchyma cell layer (d), Stone cell (e).

To generate digital expression signatures for sand pear pericarp, we used the Illumina/Solexa Genome Analyzer technology (HiSeq2000) for massively parallel sequencing by synthesis. Young fruits of 80 days after pollination (DAP) from both russet- and green-pericarp offspring of ‘Cuiguan’ × ‘Qingxiang’ (Fig. 1A and B) were sampled separately, and same amount of pericarps (Fig. 1C) from each fruit were hand dissected to construct the green- (S1) and russet-pericarp pool (S2). Briefly, the Poly(A) RNA was isolated from purified total RNA using poly-T oligo-attached magnetic beads. Following purification, the mRNA was fragmented into small pieces using divalent cations under elevated temperature, and the cleaved RNA fragments copied into first strand cDNA using reverse transcriptase and random primers. Second strand cDNA synthesis was done using DNA Polymerase I and RNaseH, the cDNA fragments processed for end repair, an addition of a single ‘A’ base, and ligation of the adapters. These products were then purified and enriched by PCR to create the final cDNA library for sequencing. In total, approximately 24 million filtered, high-quality reads with average length of 96-bp were generated from each sample. All reads of sequencing have been deposited in NIH Sequence Read Archive with RUN accession SRR925365.

### Mapping Short Reads to the Sand Pear Reference Genome

We used Tophat [44] to map the filtered reads to the sand pear reference genome (Pbr_v1.0 [51]). And 77.4% and 85.1% filtered reads of S1 and S2, respectively, were mapped. Next, we used the mapping coordinates for filtered reads that matched in the genome to extract gene information. By using the software Trinity, 2, 2046 non-redundant mapping coordinates were found to have predicted ORF and the rest 4, 101 have no. The predicted ORFs range from 150 bp to 15285 bp in length and 1236 bp on average. After removing the transfrags less than 200 bp in length, 23, 002 assemblies were deposited at DDBJ/EMBL/GenBank under the accession GALR00000000. The version described in this paper is the first version, GALR01000000.

### Gene Ontology Category Analysis in Sand Pear Pericarps

**Figure 2:**
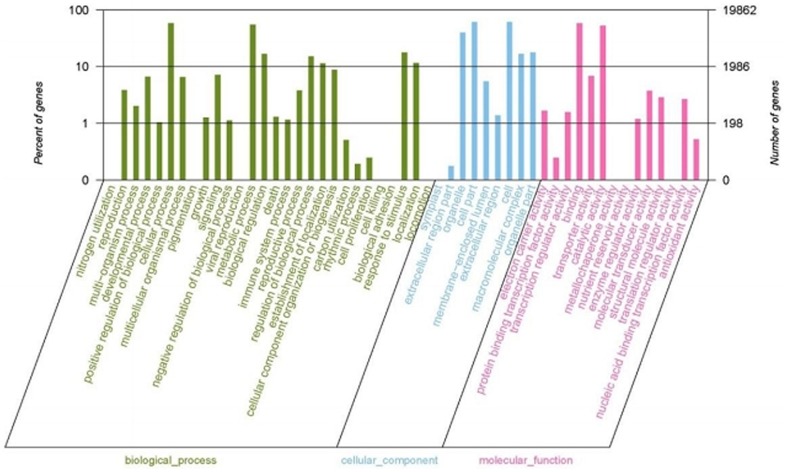
Functional categorization of the genes detected in sand pear pericarps. The functional categorization was performed using WEGO (Web Gene Ontology Annotation Plot).

In order to create a profile of gene expression in sand pear pericarps, we used WEGO (Web Gene Ontology Annotation Plot) for gene annotation analysis (Figure 2). The transcripts with and without ORF were compared to the protein non-redundant database using BlastP and BlastX, respectively, at an E-value cutoff of 1.0×10^−5^. Gene ontology (GO) terms were extracted from the best hits from BlastP or BlastX against the NR database using blast2go, and were sorted using an in-house perl scripts. WEGO analysis assigned the transcripts to three top functional categories, i.e. Cellular Component, Molecular Function, and Biological Process. As a result, a total of 19, 862 unigenes had at least one GO functional category.

In all of the three GO categories, genes with a Gene Ontology annotation were further classified into subsets. There are 28 subsets within the Biological Process category, 9 within the Cellular Component category, and 14 within the Molecular Function category (Figure 2). A closer look at these subsets showed that the majority of the unigenes are related to cellular process, metabolic process, regulation of biological process, localization, establishment of localization, and response to stimulus in the Biological Process category; organelle, cell part, cell, molecular complex and organelle part in the Cellular Component category; and binding, and catalytic activity in the Molecular Function category. The results provide a comprehensive profile for the biological synthesis and metabolic characteristics for sand pear young pericarps.

### Differential Expression and GO Enrichment of Candidate Genes

**Figure 3:**
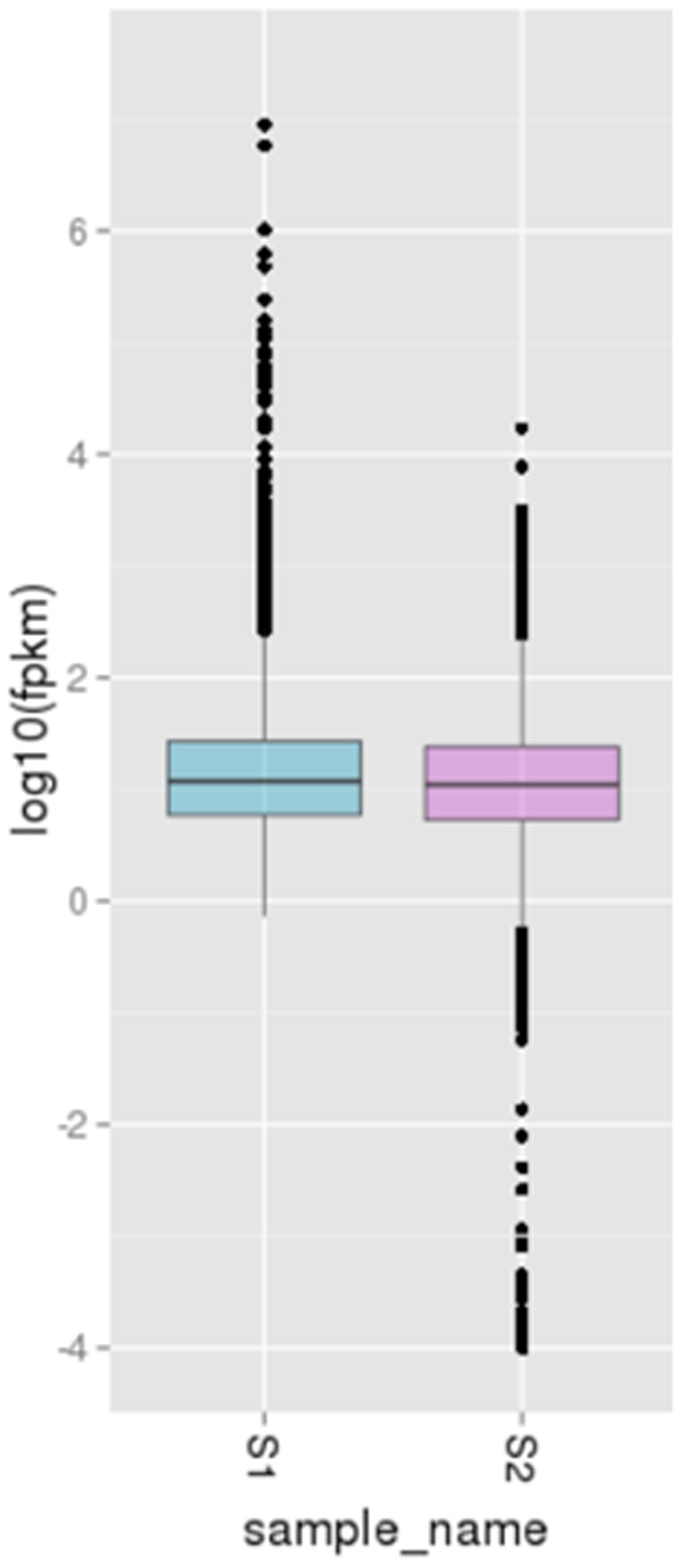
Boxplot of the log FPKM (Fragments Per Kilobase of exon per Million fragments mapped) expression values in both the green- (S1) and the russet-pericarp pool (S2) libraries of sand pear. The figure shows that the median of the expression values across the libraries being compared for differential expression are comparable.

**Figure 4:**
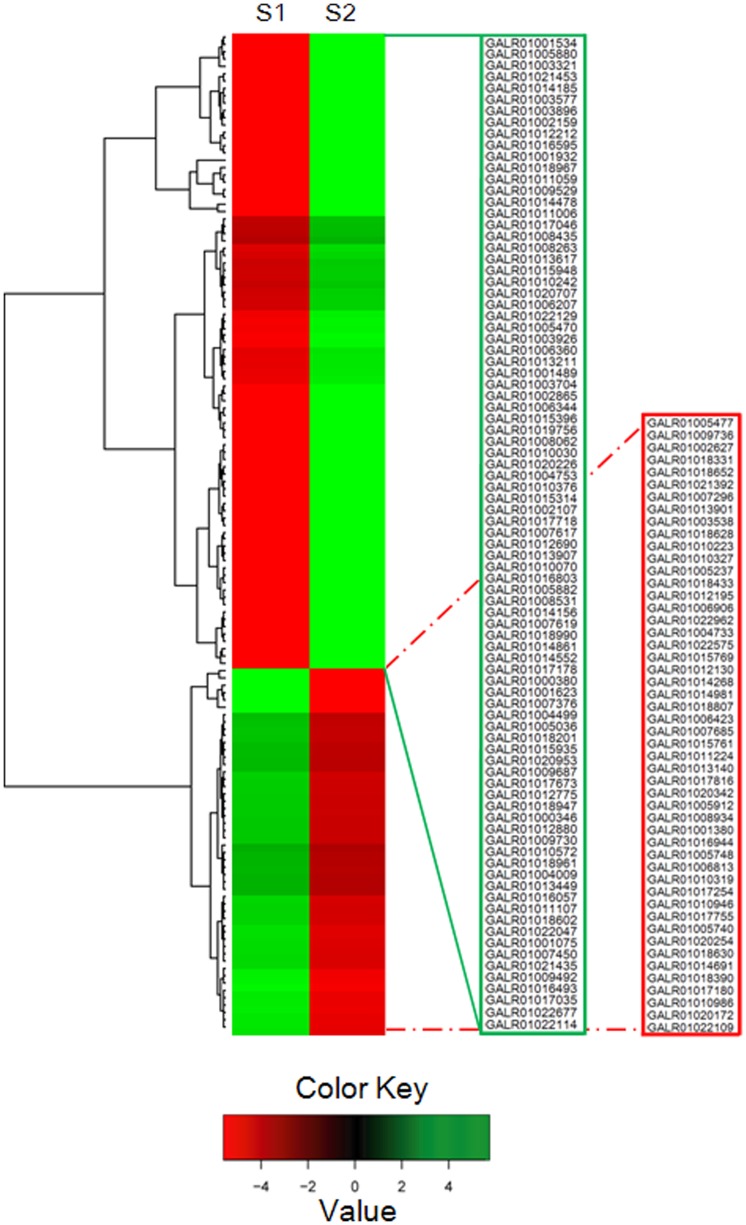
Cluster analysis of genes (137 in total) differentially expressed between the green- (S1) and the russet-pericarp (S2) pool libraries. The Cluster analysis was performed using Hierarchical cluster analysis. Rows represent differentially expressed genes, columns represent contrast groups. Green and red boxes represent genes showing lower and higher expression level, respectively. The unit of measurement used by Cufflinks to estimate transcript abundance is Fragments Per Kilobase of exon per Million fragments mapped (FPKM). Color saturation reflects magnitude of log2 expression ratio for each gene. Annotations of part genes see Table S1 in File S1.

The transcript abundance of each gene was estimated by fragments per kilobase of exon per million fragments mapped (FPKM). As shown by the box-plot distribution of the log FPKM values in Figure 3, the median and the quartile values for differential expression among the samples for comparison were almost identical. By using a very stringent cutoff for calculating differential expression as described in “Materials and Methods”, 50 genes with increased transcript abundance and 156 genes with decreased transcript abundance were identified in the S2 library compared to those in the S1 library. After removing of the genes with transfrags less than 200 bp in length, 137 genes were reserved with 50 genes with increased expression and 87 genes with decreased expression in the S2 library (Fig. 4).

One hundred and forty-nine unigenes that were differentially expressed between the S1 and S2 libraries were found to have homolog hits in the NR database, with 139 having known functional annotations. There were 123 unigenes in GO terms related to ‘cellular_component’ and ‘biological_process’, suggesting developmental and growth differentiation between the two types of pericarps (Table S1 in File S1). Several distinct patterns of gene expression related to certain pericarp pool are shown in heat-map representation (Fig. 4). GO categories corresponding to various aspects of ‘lipid metabolic processes’ were enriched with genes with divergent expressions between the two libraries. Similar phenomena were also observed in the GO categories ‘carbohydrate metabolic process’, ‘transport’ and ‘response to stress’, etc. In the case of the GO categories, such as ‘signal transduction’, ‘cell wall organization or biogenesis’, ‘cellular amino acid metabolic process’ and ‘extracellular region’, the S2 library also showed an enrichment of genes with decreased transcript abundance (Table S1 in File S1). A set of these categories were chosen for more detailed examinations.

#### i) Differential Expression of Lipid Metabolic Process-Associated Genes

**Figure 5:**
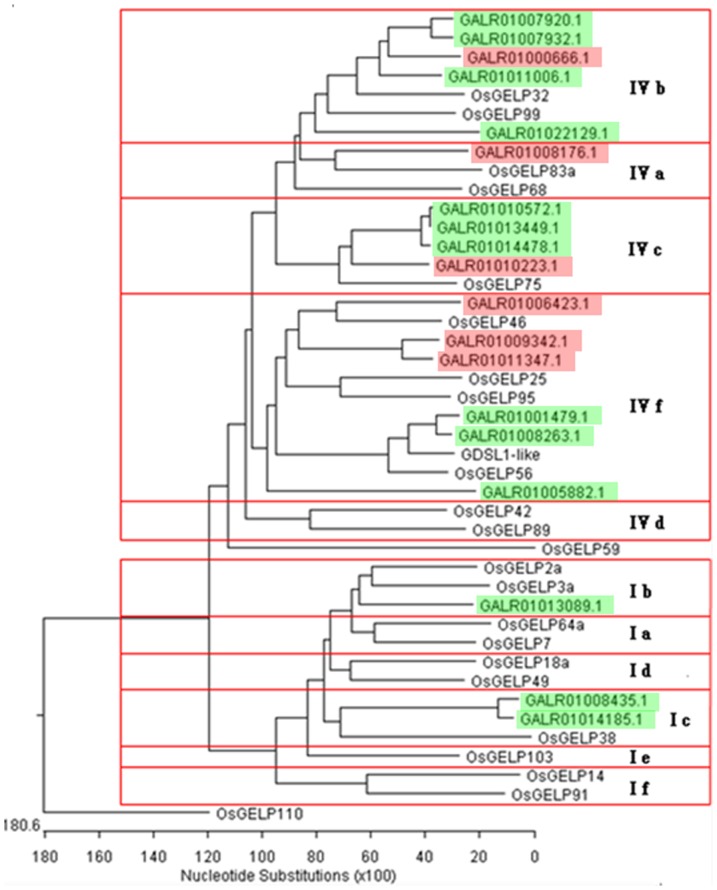
The phylogenetic relationship of the sand pear GDSL esterase/lipase genes (*GELPs*) differentially expressed between the green- (S1) and the russet-pericarp (S2) pools. The tree was constructed based on multiple sequence alignment of sand pear and rice GELP protein sequences using ClustalW program by NJ method with 1,000 bootstrap replicates. The different types of rice GELPs (Chepyshko *et al*., 2012) were used as the reference in the phylogenetic analysis. Subclades are numbered at the right part of the tree according to the rice results by Chepyshko *et al*. (2012). The sand pear GELP*s* marked in red or green show relative higher and lower transcript level in the russet pericarp pool library, comparing with that of the green pericarp pool library.

As shown in Table S2 in File S1, 36 genes associated with lipid metabolic process were detected to be differentially expressed between the S1 and S2 libraries. Among the 19 GDSL esterase/lipase genes that were differently expressed, thirteen showed decreased transcript abundance in the S2 library, while the rest showed more abundant transcripts. Phylogenetic analysis classified these GDSL genes into two groups, with the larger group consisting of 16 genes being further divided into 4 subgroups and the smaller group having 3 genes into 2 subgroups, respectively (Figure 5). Subgroup IVb and IVc each contains only one up-regulated GDSL esterase/lipase gene, but has 4 and 3 down-regulated GDSL esterase/lipase genes, respectively, in the S2 library. The 6 members in Subgroup IVf further separated into two classes, which have 3 members in each and showed increased and decreased transcript level in the S2 library, respectively. The Subgroup IVa contains only one GDSL esterase/lipase gene that showed increased expression in the S2 library. The three genes falling into the smaller group all showed decreased expression in the S2 library.

The 3-ketoacyl-coa synthase family is thought to play a role in the biosynthesis of cuticular waxes or fatty acid chains elongation. Six genes belonging to this family showed decreased transcript abundance in the S2 library. Three genes encoding putative fatty acid desaturase (GALR01006344, GALR01016595 and GALR01012775) also showed decreased expression. Some genes involved in the synthesis of wax, cutin, suberin or lignin but not assigned a GO term as ‘lipid metabolic process’ also showed decreased expression in the S2 library, for instance, the genes encoding putative glycerol-3-phosphate acyltransferase (GALR01020226), cinnamoyl-CoA reductase (GALR01020707) and BAHD acyltransferase DCR-like proteins. On the contrary, genes encoding putative cytochrome P450 (GALR01020172) and fatty acyl-CoA reductase 3 isoform 1 were found to exhibit increased expression in the S2 library.

#### ii) Differential Expression of Transport-Associated Genes

Nineteen genes encoding transport-associated proteins showed differential expression between the two types of pericarps (Table S3 in File S1). Among the nine genes encoding putative ATP-binding cassette transporters involved in cutin/suberin or lignin transport processes, six genes showed increased transcript abundance in the S2 library while the other three showed decreased transcript levels in the S2 library. There were four more genes showing relative higher transcript levels in the S2 library, which encode the putative lipid binding protein (GALR01018628), non-specific lipid-transfer protein (GALR01016944), Sec14 cytosolic factor (GALR01020254) and heavy metal transport associated protein (GALR01018630), respectively. Among the remaining genes with decreased transcripts, one gene encodes homolog of small molecule transporters (GALR01014552), and two genes encode putative Sec14 cytosolic factor (GALR01011107) and non-specific lipid-transfer protein (GALR01016493), respectively. Two genes encoding small molecule transporters also showed decreased transcripts in the S2 library.

#### iii) Differential Expression of Stress-responsive Genes

A total of six genes showed increased transcript levels in the S2 library, including four genes encoding putative peroxidases involved in phenylalanine metabolism (Table S4 in File S1). Though two genes encoding putative disease resistance response protein and major latex-like protein, respectively, showed increased transcript levels in the S2 library, three putative biotic stimulus responsive genes showed decreased transcript levels. In addition to these biotic stress responsive genes, abiotic stress responsive genes were also detected with differential expression between the two libraries. For example, the genes encoding xanthine dehydrogenase and aldehyde oxidase 4-like protein were up-regulated in the S2 library while two putative dehydration-responsive RD22 genes showed decreased expressions. Homologs of the two up-regulated genes have been associated with drought tolerance and ABA biosynthesis [52]–[54].

#### iv) Genes Involved in the Oxidation-reduction Process

A total of 32 genes falling into GO terms of ‘oxidation-reduction process’ or ‘oxidoreductase activity’ showed altered expressions between the two libraries, of which sixteen also were classified into the ‘lipid metabolic process’, ‘response to stress’ or ‘transport’ categories (Table S5 in File S1). Among the rest sixteen genes, two putative cytochrome P450 genes (GALR01005880 and GALR01010030) and one putative aldehyde dehydrogenase gene showed decreased transcript abundances in the S2 library. Genes encoding a homolog of short chain dehydrogenase/reductase (GALR01018652), uncharacterized cytochrome P450 (GALR01017816 and GALR01014691), a putative phenylcoumaran benzylic ether reductase (GALR01017755) and an alcohol dehydrogenase (GALR01015769) showed increased transcript abundance in the S2 library. But a gene encoding putative cinnamyl alcohol dehydrogenase involved in the lignin biosynthetic pathway (GALR01013211) showed decreased transcript abundance in the S2 library. Genes involved in the carbohydrate metabolism, i.e. three homologs of galactose oxidase that catalyze the sterospecific oxidation of primary alcohols to the corresponding aldehydes, and one putative NADP-dependent D-sorbitol-6-phosphate dehydrogenase (S6PDH) gene that promotes sorbitol biosynthesis, showed opposite transcriptional regulations. The remaining genes in this category included a putative 2-oxoglutarate-dependent dioxygenase gene (GALR01014156) and a putative flavonol synthase gene (GALR01012690). GALR01014156 could be involved in GA biosynthesis.

### Quantitative Real-Time-PCR Validation of Differentially Expressed Transcripts from RNA-Seq

**Figure 6:**
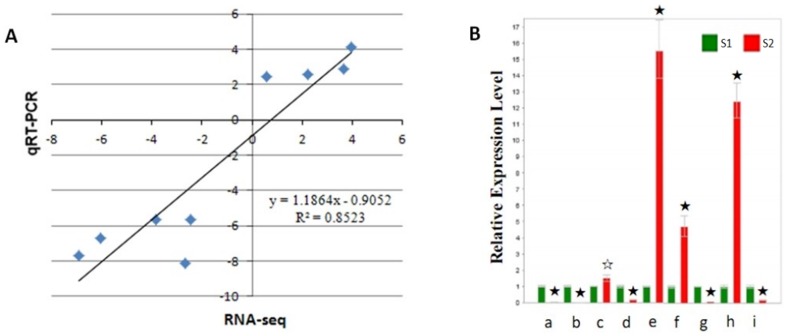
qRT-PCR validation of differentially expressed genes between the two pericarp pools of sand pear. A, Correlation of fold change analyzed by RNA-Seq platform (x axis) with data obtained using real-time PCR (y axis). B, Expression analysis of the 9 differentially expressing unigenes revealed by RNA-seq assessed through qRT-PCR. S1 and S2 represent the sand pear pericarp pools of 80 DAP the green- (S1) and the russet-pericarp fruits (S2), respectively. The expression level of these unigenes in S1 was normalized to be the same. a∼i represent the 9 differentially expressing unigenes, i.e. a, GALR01005880; b, GALR01015948; c, GALR01006906; d, GALR01012212; e, GALR01009736; f, GALR01010946; g, GALR01016595; h, GALR01020254 and i, GALR01020707, respectively (Table S1 in File S1). Error bars represent SE (n = 3). Asterisks indicate levels of significance of differential expression (t test: ☆, P<0.05; ★, P<0.01).

To validate the accuracy and reproducibility of the Illumina RNA-Seq results, a set of genes that showed altered transcript abundance between the S1 and S2 libraries were chosen for quantitative real-time (qRT)-PCR (Fig. 6). Correlation between the two methods was measured by scatterploting log2 fold changes between RNA-Seq and qRT-PCR, which is defined as DDCT (for comparative threshold cycle). As shown in Figure 6, high correlation was observed between the results from qRT-PCR and RNA-Seq, with the correlation coefficient (R2) as high as 0.85.

## Discussion

Pericarp color is a highly variable trait controlled by a relatively complex genetic mechanism. Although histogenesis and histochemical characterizations have been made in some previous researches, components and biological functions of sand pear russet pericarp in the defense and stress tolerance are largely speculated. RNA-Seq is a recently developed approach to transcriptome profiling that uses deep-sequencing technologies, which provides a far more precise measurement of levels of transcripts and their isoforms than other methods [55]. In this study, the genes differentially expressed between the green- (S1) and russet-pericarp (S2) pools of sand pear were identified with RNA-Seq on the whole genome level. In order to verify the truth of differential expression genes (DEGs), several DEGs were analyzed using qRT-PCR. One hundred and seven of the DEGs showed same expressional change with significant similarity (R2 = 0.88) in comparison with the expression data by RNA-seq from sand pear pericarp russet/green mutation (unpublished data) (Table S6 in File S1). These 107 DEGs could be used as the core genes for pericarp russet pigmentation in the next studies. Annotation of these genes suggests major differences in *Suberin and wax biosynthesis, Transport, Stress and defence, Cell wall*, and *Regulation* biological processes between the two types of sand pear pericarps. These new data will help to reveal the molecular basis of differentiation and russet pigmentation of sand pear pericarps.

Pear pericarp is made up of the cuticle lamellae, epidermal cell layer and cork cambium/cork layer from the surface to the inner side (Figure 1C; [40], [41]). Genes involved in the biosynthesis and metabolic processes of suberin, cutin, wax and lignin showing different transcript abundance between the two types of pericarps indicate variation in the biochemical composition of the cuticle and cork layer, which could lead to the differentiation in sand pear pericarp colors.

### Suberin, Cutin, Wax and Lignin Metabolic Process

The 3-ketoacyl CoA synthase (StKCS6/CER6) catalyzes the condensation of C2 units to an acyl CoA, which is the first step in VLCFA biosynthesis. Repressed expression of StKCS6/CER6 can significantly reduce the formation of suberin and wax lipidic monomers with VLCFAs [16], [56]–[60]. In our study,six 3-ketoacyl CoA synthase homologs showed repressed expression in sand pear russet pericarps(Table S2 in File S1), which indicates that the VLCFA synthesis could be attenuated in the russet pericarps as compared with that in the green pericarps. The protein HOTHEAD was revealed to catalyze the biosynthesis of long-chain alpha-, omega-dicarboxylic fatty acids and the formation of extracellular matrix [61]. The down-regulation of HOTHEAD-like gene (GALR01022575) in the S2 library also suggests repressed biosynthesis of suberin and cutin in the russet pericarps.

Microsomal delta-12 fatty acid desaturase (FAD2) and omega-3 fatty acid desaturase (FAD3) are essential enzymes in the production of polyunsaturated fatty acids [62], [63], which are substrates for cutin, suberine and wax biosynthesis (PATHWAY: ko00073). CYP 77 and CYP 86 have been known to catalyze the cutin and suberine biosynthesis with polyunsaturated fatty acids [64]. The repressed expression of sand pear FAD2, FAD3 and CYP homologs (Table S2 in File S1) suggests the biosynthesis of cutin, suberine and wax be repressed in sand pear russet pericarps. However, another CYP 86 homolog (GALR01020172) showed enhanced expression in the S2 library and its biological significance remains to be elucidated.

Omega-hydroxypalmitate O-feruloyl transferase is responsible for synthesizing suberin aromatics in Arabidopsis [65]. In our study, three Omega-hydroxypalmitate O-feruloyl transferase homologs (GALR01016057, GALR01007617 and GALR01018990) showed decreased transcript abundance in the S2 library, which indicates that the synthesis of suberin could be repressed in sand pear russet pericarps. Furthermore,two putative BAHD acyltransferase DCR-like genes (GALR01012690 and GALR01017755), which was shown to be required for cutin polyester formation [66], also were down-regulated in the S2 library.

Previous studies revealed that GDSL (Gly-Asp-Ser-Leu) esterases/lipases are preferentially expressed in epidermal cells of the pericarp, close to the cuticular membranes [67]–[71]. The expression of an Arabidopsis GDSL-lipase gene is up-regulated by WIN1/SHN1, a transcription factor that regulates cutin deposition [17], [72]. Girard *et al*. [73] reported that tomato GDSL1 is required for cutin deposition in the fruit cuticle. In this study, 19 GDSL esterase/lipase genes showed significantly different expression levels between the S1 and S2 libraries. Decreased transcript abundance of the tomato GDSL1 orthologs (GALR01008263 and GALR01011059) in the S2 library also suggests repression of cutin deposition in sand pear russet pericarps. GDSL esterases/lipases belong to a newly discovered subclass of lipolytic enzymes with multifunctional properties [74]. The differentially expressed GDSL esterase/lipase genes between the S1 library and the S2 library were further divided into multiple sub-families in the phylogenetic analysis (Figure 5), based on their distinct protein structures [74]. More work is needed to reveal the specific biological functions of individual GDSL members.

Severe suppression of cinnamoyl-CoA reductase (CCR) almost halved lignin content in *Pinus radiata* tracheary element (TE) system and substantially promoted pyrolytic break-down of cell wall polysaccharides [75]. Down-regulated expression of the CCR homolog (GALR01020707) in the S2 library therefore could change the biosynthesis pathway of lignin in pericarp certain cells and contribute to form bark and bark-related compounds in russet pericarps. Previous data have shown that manipulation of the monolignol pathway in pine can enhance the formation of bark and bark-related compounds in woods [76]. Cinnamyl alcohol dehydrogenase (CAD) is a multifunctional enzyme that regulates the last step of lignin biosynthesis [77]–[79]. Silencing two CAD genes in Nicotiana attenuata resulted in red-pigmented stems, reflecting blocked lignification [80]. As such, the down regulation of the CAD homolog (GALR01015769) in the S2 library may contribute to the pigmentation of russet pericarps in sand pear. However, a previous study reported that the content of lignin in sand pear russet pericarps is higher than that in the green pericarps [81]. Kaur *et al*. [80] also showed that environmental stresses could allow CAD-deficient Nicotiana attenuata plants to accumulate more various phenylpropanoids in stems, delay and restrict spread of red stem pigmentation, and attain wild-type-comparable total lignin contents. Therefore, a CAD-independent pathway responsive to environmental stresses for lignin biosynthesis may exist in plants.

The ATP-binding cassette (ABC) transporters mediate MgATP-energized transmembrane transport and/or regulate other transporters [82]. The role of PM-localized ABC transporters was recently identified to be involved in cutin/suberin and lignin transport processes. Though most of the over 100 putative ABC transporter genes detected in sand pear pericarp pools showed similar expression levels between the S1 library and the S2 library, nine of them showed distinct transcript abundances between the two libraries (Table S3 in File S1). This indicates that the transport system of cutin/suberin and lignin may also work differentially between the russet pericarps and the green pericarps. Further studies are required to reveal the mechanisms behind the differential expression of these ABC transporters and their roles in the formation of sand pear russet and green pericarps.

### Stress Responsiveness

Since cuticle and cork layer protect plant tissues against environmental stresses, change of their components in the pericarp may affect the fruit resistance to biotic and abiotic stresses. Indeed, significant expression differences of several disease resistance responsive genes were detected between the two types of pericarps. Peroxidases are known to catalyze the biosynthesis of plant phenylpropanoids (KEGG Reference pathway: ko00940), which are important to protect plants against biotic stresses [83]–[85]. Four peroxidase gene homologs (GALR01018390, GALR01015761, GALR01007685 and GALR01011224, Table S4 in File S1) were detected with increased transcript abundance in the S2 library, consistent with the result from Li *et al*. [81] that the peroxidase activity in russet pericarps was significantly higher than in green pericarps. High peroxidase activity was also supposed to be correlated with the dark brown pigmentation of pear pericarps [86]. The aquaporin-1 (AQP1) water channel protein is known to facilitate the rapid movement of water across cell membranes [87]–[89]. In this study, a homolog of aquaporin-1 gene showed decreased transcript abundance in the S2 library. As we have previously shown, decreased accumulation of suberin/cutin/wax on the surface of sand pear russet pericarps would affect the transpiration rate; the down-regulated expression of AQP1 in pericarps could hinder the fruit surface transpiration and reduce the fruit water loss. Apart from these regulative genes, several putative biotic/abiotice stress tolerance genes (such as GALR01017718, GALR01011224 and GALR01015761, Table S1 in File S1) also showed decreased transcript abundance in the S2 library. The differential expression of these stress responsive genes reflects the variability in the mechanisms of sand pear biotic and abiotic stress tolerance, associated with sand pear pericarp color differentiation. It also indicates new members in the gene pool for sand pear stress tolerance. In addition to the biological processes discussed above, pericarp cell wall development could also be differentiated between the russet pericarps and the green pericarps. AtMYB106 was revealed to function as a repressor of epidermal cell outgrowth [90]. Two AtMYB106 homologs (GALR01014861 and GALR01006360) showed decreased transcript abundance in the S2 library. A gene encoding putative endo-1, 4-beta-glucanase associated with cell wall thickening [91], and a gene (GALR01013907) encoding putative extensin required for the self-assembly of plant cell wall [92] were also detected with repressed expressions in the S2 library.

## Supporting Information

File S1 **Supporting Information. Table S1.** Annotation to part candidate genes differentially expressed between the green- (S1) and the russet-pericarp pool (S2). Annotation source: goatools (https://github.com/tanghaibao/goatools). The extent of differential expression is measured in terms of fold change. **Table S2.** Sand pear lipid-metabolic-process related genes differentially expressed between the green- (S1) and the russet-pericarp pool (S2). The extent of differential expression is measured in terms of fold change. **Table S3.** Sand pear transport related genes differentially expressed between the green- (S1) and the russet-pericarp pool (S2). The extent of differential expression is measured in terms of fold change. **Table S4.** Sand pear stress-responsive genes differentially expressed between the green- (S1) and the russet-pericarp pool (S2). The extent of differential expression is measured in terms of fold change. **Table S5.** Sand pear oxidation-reduction process-related genes differentially expressed between the green- (S1) and the russet-pericarp pool (S2). The extent of differential expression is measured in terms of fold change. Genes with accession numbers showed in bold have been list in GO terms of ‘lipid metabolic process’, ‘transport’ or ‘response to stress’. **Table S6.** Differential expression genes (DEGs) verified by RNA-Seq data of russet/green mutants (unpublished).
(XLS)
